# A novel nonsense mutation in androgen receptor confers resistance to CYP17 inhibitor treatment in prostate cancer

**DOI:** 10.18632/oncotarget.14296

**Published:** 2016-12-27

**Authors:** Dong Han, Shuai Gao, Kevin Valencia, Jude Owiredu, Wanting Han, Eric de Waal, Jill A Macoska, Changmeng Cai

**Affiliations:** ^1^ Center for Personalized Cancer Therapy, University of Massachusetts Boston, Boston, Massachusetts 02125, USA; ^2^ Hematology-Oncology Division and Cancer Center, Beth Israel Deaconess Medical Center and Harvard Medical School, Boston, Massachusetts 02215, USA; ^3^ Biology Department, University of Suffolk, Boston, Massachusetts 02108, USA

**Keywords:** prostate cancer, androgen receptor, androgen receptor mutation, CYP17 inhibitor, abiraterone

## Abstract

The standard treatment for prostate cancer (PCa) is androgen deprivation therapy (ADT) that blocks transcriptional activity of androgen receptor (AR). However, ADT invariably leads to the development of castration-resistant PCa (CRPC) with restored activity of AR. CRPC can be further treated with CYP17 inhibitors to block androgen synthesis pathways, but most patients still relapse after a year of such treatment. The mechanisms that drive this progression are not fully understood, but AR activity, at least in a subset of cancers, appears to be restored again. Importantly, *AR* mutations are more frequently detected in this type of cancer. By analyzing tumor biopsy mRNA from CRPC patients who had developed resistance to CYP17 inhibitor treatment, we have identified a novel nonsense mutation (Q784*) at the ligand binding domain (LBD) of AR, which produces a C-terminal truncated AR protein that lacks intact LBD. This AR-Q784* mutant is transcriptionally inactive, but it is constitutively expressed in the nucleus and can bind to DNA in the absence of androgen. Significantly, our results show that AR-Q784* can heterodimerize with, and enhance the transcriptional activity of, full-length AR. Moreover, expressing AR-Q784* in an AR positive PCa cell line enhances the chromatin binding of endogenous AR and the recruitment of p300 coactivator under the low androgen condition, leading to increased cell growth. This activity of AR-Q784* mimics the function of some AR splice variants, indicating that CYP17 inhibitor treatment in CRPC may select for LBD-truncated forms of AR to restore AR signaling.

## INTRODUCTION

Prostate cancer (PCa) is the second leading cause of cancer mortality in American men and is dependent on the activity of androgen receptor (AR) for its initiation and progression. AR is a nuclear receptor transcription factor and is activated by androgen ligands (testosterone and its more potent form dihydrotestosterone, also called DHT). The AR protein contains a N-terminal transactivation domain, a DNA binding domain (DBD), a hinge region, and a C-terminal ligand binding domain (LBD). Upon agonist ligand stimulation, AR homodimerizes and translocates from the cytoplasm into the nucleus, where it can bind to specific DNA regions (called androgen response elements or AREs), to regulate the transcription of androgen responsive genes such as prostate-specific antigen (*PSA*). The standard treatment for PCa is to block transcriptional activity of androgen receptor using androgen deprivation therapy (ADT), which invariably results in the development of a more aggressive form of cancer (called castration-resistant PCa or CRPC) with restored activity of AR [1, 2]. CRPC can be further treated with more intensive androgen ablation treatments, including CYP17 inhibitors (abiraterone or ketoconazole) to block the androgen synthesis pathways and more potent AR antagonists (such as enzalutamide) [3–6]. Most CRPC patients still relapse after a year of such treatment, and AR activity appears to be restored again in at least a subset of the cancers [7].

Several mechanisms reported previously by ourselves and others may contribute to the reactivation of AR in CYP17 inhibitor-resistant PCa, including increased intratumoral steroid synthesis [8, 9], selection for progesterone-driven T878A mutation of AR [10], and activation of ErbB2 pathway that stabilizes AR protein [11, 12]. Another important mechanism contributing to the resistance may be increased expression of AR splice variants that lack the intact LBD and can be constitutively activated without ligand stimulation [13–17]. The most important *AR* splice variant appears to be *AR-V7* (or *AR3*), which can be constitutively activated without androgen stimulation and has increased expression under conditions of CRPC and abiraterone treatment (or enzalutamide treatment) [18–20]. Recent studies also indicate that the major function of AR-V7 might be through enhancing transcriptional activity of full-length AR (AR-FL) in the low androgen environment through the formation of an AR-V7/AR-FL heterodimer [21–23]. Nevertheless, it is conceivable that intensive ADTs may select for the expression of AR splice variants, which may lead to the reactivation of AR in CRPC.

In this study, we have characterized a novel nonsense AR-Q784* mutation identified in a CRPC patient who had been resistant to the treatment of ketoconazole, a CYP17 inhibitor. This point mutation converts glutamine to stop codon at amino acid 784 and creates a C-terminal truncated AR protein. As expected, AR-Q784* mutant produces an AR protein with partial truncation of LBD, which localizes in the nucleus and can bind to AREs without ligand stimulation. Surprisingly, AR-Q784* does not elicit transcriptional activity when overexpressed alone in AR negative cell lines. However, when co-expressed with AR-FL, this mutant AR can enhance AR-mediated transcriptional activity through possible heterodimerization with AR-FL in PCa cells. When AR-Q784* was overexpressed in an AR-positive PCa cell line, it enhanced the transcriptional activity of endogenous AR-FL particularly under low androgen conditions through stabilizing DNA binding of AR-FL and strengthening the AR recruitment of p300 coactivator, resulting in increased proliferation of PCa cells. These findings demonstrate that PCa cells under CYP17 inhibitor treatments may select for the expression of LBD-truncated ARs through a novel class of point mutations. In addition, our results suggest that expression of these AR mutants may function to enhance the activity of AR-FL under very low androgen conditions in CRPC treated with CYP17 inhibitors.

## RESULTS

### AR-Q784* mutant does not confer transcriptional activity

**Figure 1:**
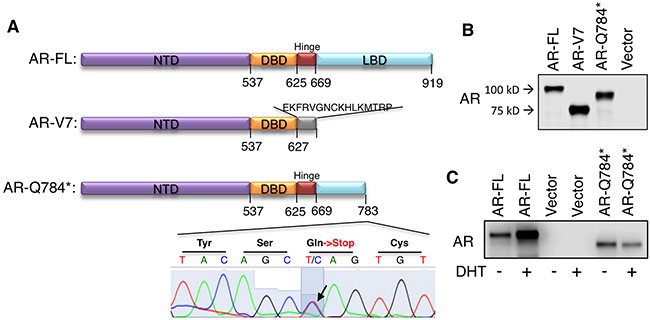
AR-Q784* produces a C-terminal truncated form of AR protein **A**. Predicted protein structure of AR-FL, AR-V7, or AR-Q784*. **B**. Immunoblotting for AR (using antibody against N-terminal region) in COS-7 cells transfected with AR-FL, AR-V7, AR-Q784*, or empty vector. **C**. Immunoblotting for AR (N-terminal) in PC-3 cells transfected with AR-FL, empty vector, or AR-Q784*, and treated with vehicle or 10nM DHT (24h).

LBD mutations of *AR*, including progesterone-driven *AR-T878A*, are more frequent in CRPC and in CRPC resistant to more intensive ADTs [10, 24]. To identify additional *AR* LBD mutations in CYP17A1 inhibitor treated tumors, we examined a set of CRPC samples obtained from CT-guided bone marrow biopsies from patients treated with high dose ketoconazole (a CYP17A1 inhibitor) and dutasteride (5α-reductase inhibitor), which were used to maximally suppress androgen synthesis [6, 8]. In a patient with relapse, we detected significant expression of a novel nonsense mutation (3465C->T, Q784*) from his tumor sample (Figure 1A). Both mutant *AR* and wildtype *AR* are expressed in this sample, but whether they were coexpressed in the same tumor cell is unknown. *PSA* was also highly expressed (data not shown) but the expression of *CYP17A1* was relatively low [8] (sample # P5). This nonsense mutation at exon 6 of the *AR* gene produced a C-terminal truncated form of AR, which included only a fraction of the LBD (Figure 1A). In contrast, the *AR-V7* splice variant produces a protein completely devoid of LBD, but consists of an additional C-terminal amino acid sequence translated from its cryptic exon 3 [14, 15]. To examine the function and activity of this novel *AR* mutation, we performed site-direct mutagenesis (substituting C with T at position 3465) in wildtype *AR* to generate the AR-Q784* mutant. As seen in Figure 1B, in COS-7 cells (an AR-negative cell line) AR-Q784* was translated to a protein that was smaller in size than wildtype AR (AR-FL), but larger than AR-V7. Unlike AR-FL, DHT had no stimulatory effect on AR-Q784* protein expression in PC-3 cells (an AR-negative PCa cell line), supporting that AR-Q784* is deficient in ligand binding ability (Figure 1C).

**Figure 2:**
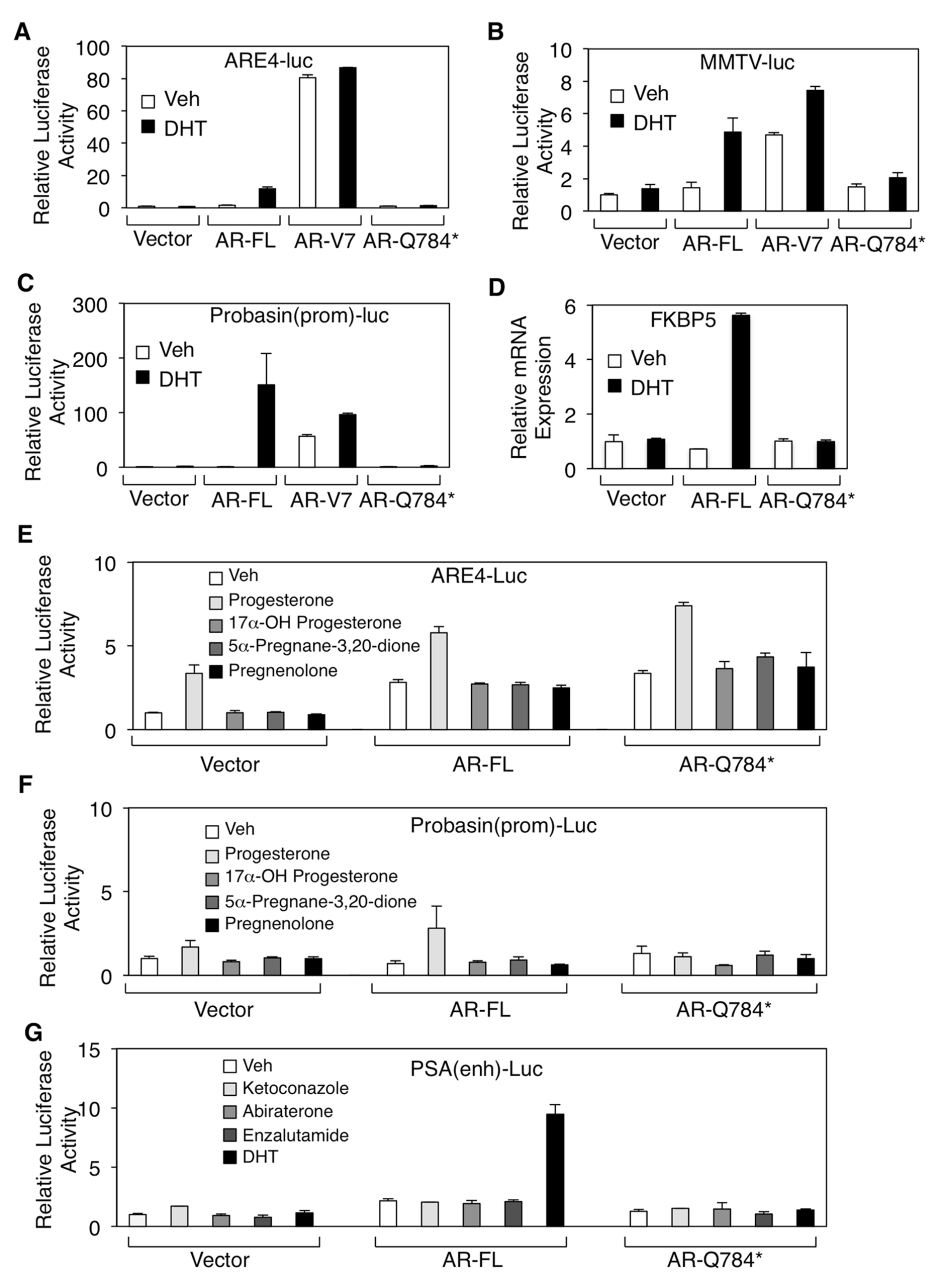
AR-Q784* mutant is deficient for androgen stimulated transcription activity **A, B, C**. Dual-luciferase reporter assays were carried out in PC-3 cells transfected with empty vector, AR-FL, AR-V7, and AR-Q784* using various androgen-regulated reporters driven by (A) ARE4, (B) *MMTV* promoter, or (C) *Probasin* promoter, and treated with vehicle or 10nM DHT (24h). Reporter activity was normalized to the cotransfected CMV-*Renilla*-Luc. **D**. Endogenous *FKBP5* mRNA expression was examined by qRT-PCR in PC-3 cells transfected with empty vector, AR-FL, or AR-Q784*, and then treated with 10nM DHT (24h). **E, F, G**. Dual-luciferase reporter assays were performed in COS-7 cells transfected with empty vector, AR-FL, or AR-Q784*, and treated with the indicated small molecules using reporters driven by (E) ARE4, (F) *Probasin* promoter, or (G) *PSA* enhancer.

Because AR-V7 is constitutively activated in prostate cancer cells, we next determined whether AR-Q784* could also confer transcriptional activity in the absence of ligand stimulation. Surprisingly, the expression of AR-Q784* in PC-3 cells failed to stimulate reporter activities driven by androgen response elements (artificial ARE, *MMTV* promoter, or *probasin* promoter) or activate the transcription of endogenous *FKBP5* gene, a well-studied androgen-regulate gene, in either the absence or presence of androgen treatments (Figure 2A–2D). Previous studies have shown that the activity of the AR-T878A mutant in abiraterone-resistant PCa cells is driven by upstream CYP11A1-dependent intraturmoral progesterone synthesis [8, 10]. Therefore, we sought to determine if any progesterone-related androgen precursors (progesterone, 17α-OH progesterone, 5α-pregnane-3,20-dione, or pregnenolone) can activate AR-Q784*. As seen in Figure 2E and 2F, none of those androgen precursors was able to stimulate substantial transcriptional activity of AR-Q784*. A recent study suggested that the metabolites of abiraterone could function as agonists in some contexts [25]. Therefore, we examined whether ketoconazole or abiraterone could induce transcriptional activity of AR-Q784*. As seen in Figure 2G, none of the CYP17 inhibitors or enzalutamide (a potent AR antagonist) stimulated the reporter activity driven by the androgen-regulated *PSA*-enhancer in AR-Q784* transfected COS-7 cells. Collectively, these results suggest that the partial LBD truncation in AR-Q784* impairs its ability to activate expression of AR-regulated genes.

### AR-Q784* localizes in nucleus and binds to chromatin independent of androgen stimulation

**Figure 3:**
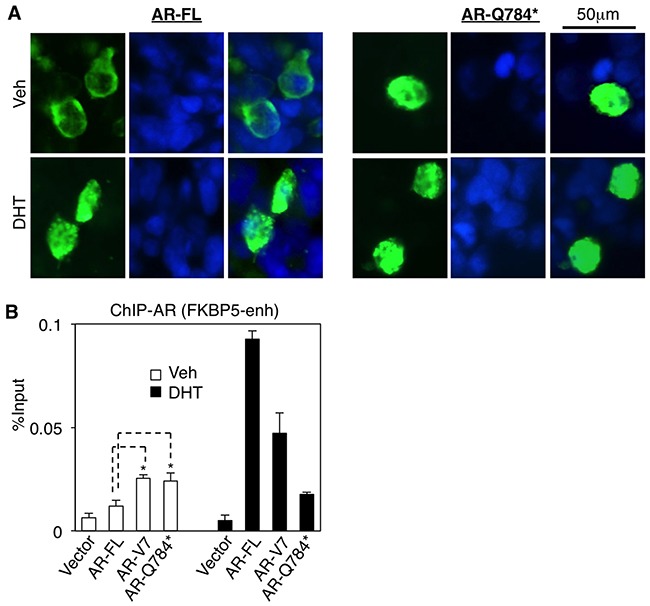
AR-Q784* localizes in nucleus and binds to chromatin independent of androgen stimulation **A**. Immunofluorescence staining for AR (N-terminal) (green) in COS-7 cells transfected with AR-FL or AR-Q784* and treated with 10nM DHT for 24h. Nuclear compartment was determined by DAPI staining (blue). **B**. ChIP-qPCR for measuring AR binding (N-terminal) at an *FKBP5* enhancer in PC-3 cells transfected with empty vector, AR-FL, AR-V7, or AR-Q784*, and treated with vehicle or 10nM DHT (4h).

**Figure 5:**
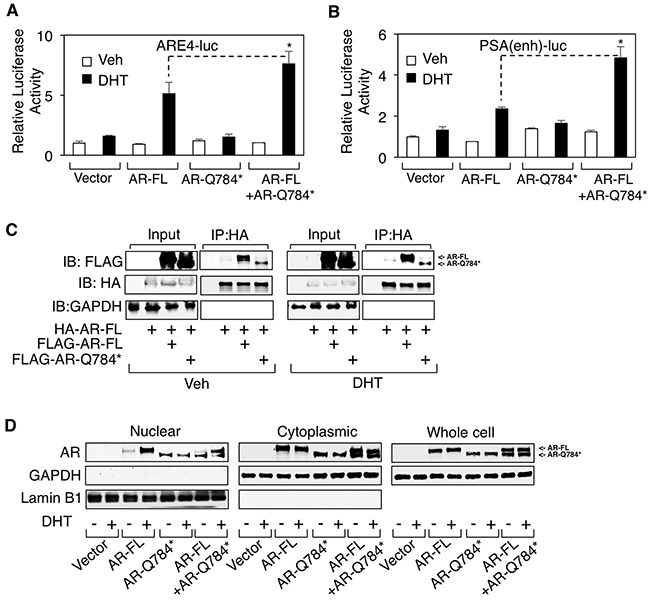
AR-Q784* heterodimerizes with AR-FL and enhances its transcriptional activity **A, B**. Dual-luciferase reporter assays were carried out in PC-3 cells transfected with empty vector, AR-FL, AR-Q784*, or AR-FL+AR-Q784* using androgen-regulated reporters driven by (A) ARE4 or (B) *PSA* enhancer, and treated with vehicle or 10nM DHT (24h). **C**. Immunoprecipitation for HA in COS-7 cells cotransfected with HA-tagged AR-FL plus FLAG-tagged AR-FL or AR-Q784*, and treated with vehicle or 10nM DHT (24h), followed by immunoblotting for FLAG or HA. **D**. COS-7 cells transfected with empty vector, AR-FL, AR-Q784*, or AR-FL plus AR-Q784*, and treated with vehicle or DHT were fractionated into nuclear and cytoplasmic extracts, followed by immunoblotting for AR (N-terminal). GAPDH is a loading control for whole cell extracts and cytoplasmic fraction. Lamin B1 is a loading control for nuclear fraction.

AR-Q784* has an intact nuclear localization signal (NLS) [26, 27] and intact DBD domain, therefore we next determined whether AR-Q784* can translocate to nucleus and bind to chromatin. As seen in Figure 3A, AR-Q784* (green) localized in both cytoplasm and nucleus with and without stimulation of DHT. However, AR-FL largely resided in the cytoplasm in the absence of androgen treatment but translocated into the nucleus upon DHT stimulation (similar result was also seen in Figure 5D). This subcellular localization of AR-784* is similar to AR-V7, which is constitutively expressed in the nucleus [21]. We then performed a ChIP assay in AR-Q784* transfected PC-3 cells to determine whether AR-Q784* can still bind to AREs without androgen stimulation. As shown in Figure 3B, both AR-Q784* and AR-V7 can constitutively bind to the *FKBP5*-enhancer region in the presence and absence of DHT with similar binding affinity. In contrast, the binding of AR-FL was much weaker in the absence of androgen but was markedly induced by DHT stimulation. Overall, these data showed that AR-Q784* is constitutively expressed in the nucleus and can bind to AREs without androgen stimulation.

**Figure 4:**
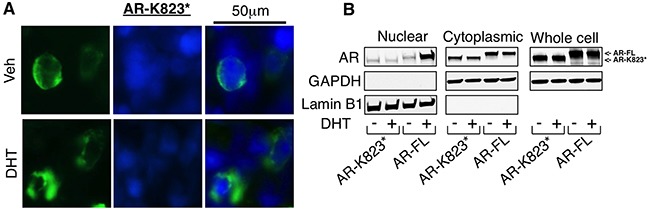
Loss of intact NES contributes for the nuclear localization of AR-Q784* **A**. Immunofluorescence staining for AR (N-terminal) (green) in COS-7 cells transfected with AR-K823* and treated with 10nM DHT for 24h. Nuclear compartment was determined by DAPI staining (blue). **B**. COS-7 cells transfected with AR-FL or AR-K823* and treated with vehicle or DHT were fractionated into nuclear and cytoplasmic extracts, followed by immunoblotting for AR (N-terminal). GAPDH is a loading control for whole cell extracts and cytoplasmic fraction. Lamin B1 is a loading control for nuclear fraction.

The nuclear expression of AR-Q784* may be also due to partial loss of nuclear export signal (NES) that is within LBD of AR (amino acid 742-817) [28]. This NES is active in absence of ligand and repressed upon ligand binding, and is dominant over NLS when hormone ligand is absent. To further determine the impact of NES on regulating AR-Q784* subcellular localization, we generated another nonsense mutation of *AR* at lysine 823 (AR-K823*), which produces a C-terminal truncated AR that retains both NLS and NES. In contrast to the constitutively nuclear localization of AR-Q784*, AR-K823* is largely excluded from nucleus and retained in cytoplasm regardless of hormone stimulation(Figure 4A and 4B). These results indicated an essential role of NES in regulating the subcellular localization of AR mutants/variants.

### AR-Q784* heterodimerizes with AR-FL and enhances its transcriptional activity

Our results indicate that this LBD-disrupted AR mutant is deficient in its transcriptional activity despite its ability to bind to chromatin even in absence of androgen stimulation (see Figure 2 and 3). However, previous studies on AR splice variants suggest that many AR variants may function to enhance full length AR activity in CRPC cells [21–23]. Thus, we hypothesized that AR-Q784* may play a role in supporting wildtype AR activity in CRPC cells. To test this hypothesis, we co-transfected AR-FL and AR-Q784* into PC-3 cells to determine if AR-Q784* could enhance the activity of AR-FL. Indeed, coexpression of AR-Q784* with AR-FL significantly increased reporter activities (driven by ARE4 or *PSA*-enhancer) compared to AR-FL alone (Figure 5A and 5B), suggesting that AR-Q784* may function as a coregulator of AR-FL to activate transcription. As previous reports showed that AR-V7 can heterodimerize with AR-FL to stimulate transcription [23], we next sought to determine if AR-Q784* and AR-FL can similarly heterodimerize to form a protein complex. To test this, we co-transfected COS-7 cells with HA-tagged AR-FL in combination with either FLAG-tagged AR-FL or FLAG-tagged AR-Q784*, treated the transfected cells with vehicle or DHT, then performed an HA pull-down assay. As seen in Figure 5C, we detected an interaction between HA- and FLAG-tagged AR-FL proteins and this interaction may be further increased upon DHT stimulation. Importantly, AR-Q784* was also capable of interacting with AR-FL regardless of androgen treatment. To determine if this heterodimerization can influence the subcellular localization of AR-FL, we next performed a cell fractionation assay in COS-7 cells transfected with AR-FL and AR-Q784*. However, we did not observe any substantial effect on androgen-stimulated nuclear translocation of AR-FL by coexpressing AR-Q784* (Figure 5D), indicating that enhanced activity of AR-FL was not due to increased nuclear translocation by forming heterodimer with AR-Q784*. Nevertheless, these results showed that AR-Q784* can heterodimerize with AR-FL and this interaction may enhance the transcriptional activity of AR-FL.

### AR-Q784* enhances endogenous AR-FL transcription activity in PCa cells stimulated by low levels of androgen

**Figure 6:**
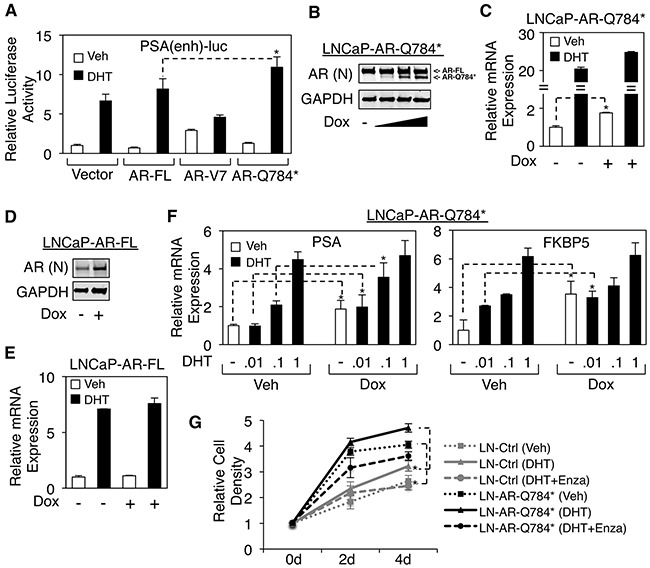
AR-Q784* enhances the transcription activity of endogenous AR-FL in PCa cells stimulated by low levels of androgen **A**. Dual-luciferase reporter assays were carried out in LNCaP cells transfected with empty vector, AR-FL, AR-V7, or AR-Q784* using *PSA* enhancer-driven reporter, and treated with vehicle or 10nM DHT (24h). **B, D**. Immunoblotting for total AR expression (N-terminal) in LNCaP cells stably infected by lentiviral vectors containing doxycycline-inducible (B) AR-Q784* (LNCaP-AR-Q784*) or (D) AR-FL (LNCaP-AR-FL), and treated with doxycycline (0, 0.02, 0.1, or 0.5μg/ml) for 2d. (**C, E**) qRT-PCR for the mRNA expression of endogenous *PSA* in (C) LNCaP-AR-Q784* or (E) LNCaP-AR-FL cells pretreated with doxycycline for 2d and then stimulated by 10nM DHT for 24h. **F**. qRT-PCR for *PSA* expression in LNCaP-AR-Q784* pretreated with doxycycline for 2d and then stimulated by 0-1nM DHT for 24h. **G**. Flow cytometry analysis (PI staining) for measuring the proliferation of the LNCaP stable cell line with empty vector or LNCaP-AR-Q784* treated with vehicle, 0.1nM DHT, or 0.1nM DHT+10μM enzalutamide for 0-4d. Both control cell line and LNCaP-AR-Q784* cell line were pretreated with 0.1μg/ml doxycycline.

Since we demonstrated that AR-Q784* can enhance AR-FL transcriptional activity in PC-3 cells, we next sought to understand how this transcriptional enhancement function of AR-Q784* can contribute to reactivating AR signaling in CRPC cells under low androgen conditions. For this study, we used an androgen-sensitive LNCaP PCa cell line as the model system. Although LNCaP cells express a full length AR with a point mutation (T878A) that can be activated by progesterone, this mutant AR behaves similarly to the wildtype AR when stimulated by testosterone or DHT ligands [8]. Moreover, unlike many other PCa cell lines LNCaP cells do not express any detectable level of AR splice variants, which would compete with or interfere the activity of AR-Q784*. In LNCaP cells, the reporter activity driven by *PSA* enhancer can be induced by DHT treatment through endogenous AR and the overexpression of wildtype AR modestly increased this endogenous AR activity (Figure 6A). Interestingly, while expression of AR-V7 constitutively drove reporter activity in the absence of androgen, it actually decreased endogenous AR activity in the presence of DHT. In contrast, expression of AR-Q784* enhanced the DHT-stimulated activity of endogenous AR and this activity was also higher than that observed upon overexpression of AR-FL.

To more accurately assess the role of AR-Q784* on endogenous AR signaling, we established an LNCaP cell line that stably expresses doxycycline inducible AR-Q784* mutant by lentiviral infection (LNCaP-AR-Q784*) (Figure 6B). While AR signaling (measured by *PSA* mRNA transcription) was only modestly increased by expressing AR-Q784* upon high-level DHT (10nM) stimulation, the basal activity of AR was significantly enhanced over 2-fold (Figure 6C). In contrast, short-term induction of wildtype AR did not significantly affect androgen-induced *PSA* expression (Figure 6D and 6E), consistent with the result from the reporter assay (see Figure 6A). Based on these observations, we hypothesized that AR-Q784* may sensitize endogenous full-length AR to lower dose of androgens. To test this, we treated LNCaP-AR-Q784* cells with a range of low-dose DHT. As seen in Figure 6F, the androgen-regulated expression of *PSA* and *FKBP5* were enhanced by the presence of 0-0.1nM DHT but not by 1nM DHT, indicating that AR-Q784* may enhance the activity of AR-FL stimulated by low level of androgen ligands. Furthermore, we examined if AR-Q784* could increase PCa cell proliferation under low androgen stimulation. As shown in Figure 6G, the control LNCaP cells (LN-Ctrl) showed DHT-stimulated (0.1nM) growth, which can be fully prevented by treating cells with enzalutamide. Importantly, the LNCaP-AR-Q784* cells showed increased proliferation in either absence or presence of DHT (0.1nM) compared with LNCaP-Ctrl cells. Interestingly, enzalutamide treatment on LNCaP-AR-Q784* not only blocked DHT-induced cell proliferation but also further decreased growth in the levels lower than that of the vehicle treated cells, indicating that enzalutamide is capable to block the growth promoting effect from the AR-FL/AR-Q784* heterodimer. Together, these results highly support our hypothesis that AR-Q784* functions to sensitize full-length AR to the stimulation of low-level androgens in CRPC cells.

### AR-Q784* stabilizes AR-FL chromatin binding and enhances the recruitment of p300 coactivator

**Figure 7:**
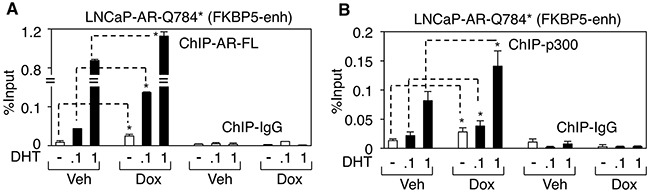
AR-Q784* stabilizes AR-FL chromatin binding and enhances the recruitment of p300 coactivator **A, B**. ChIP-qPCR for measuring (A) AR-FL binding (using antibody against C-terminal region) or (B) p300 binding at an FKBP5 enhancer in LNCaP-AR-Q784* cells pretreated with vehicle or doxycycline and then stimulated by DHT (0, 0.1 or 1 nM) for 4h.

We next sought to determine the molecular basis for the enhanced AR signaling in LN-AR-Q784* cells. To accomplish this, we examined the impact of the AR-Q784* mutant on chromatin binding of AR-FL and its coactivator recruitment. ChIP assays on full-length AR (antibody against C-terminal region of AR) and p300 (a well studied AR coactivator) were performed in doxycycline treated LN-AR-Q784* cells and their interactions with the *FKBP5*-enhancer were examined. As seen in Figure 7A, chromatin binding of AR-FL was markedly increased by coexpression of AR-Q784*, particularly in 0.1nM DHT treated cells. Moreover, increased AR binding clearly resulted in enhanced recruitment of p300 (Figure 7B), which is a hallmark of transcription activation. Overall, this study on AR-Q784* mutant in LNCaP cells clearly demonstrated its function as a coregulator of AR-FL to enhance its transcriptional activity.

## DISCUSSION

While *AR* mutations are relatively rare in untreated hormone-dependent PCa, they are more frequently detected in CRPC [24], suggesting that ADT may select for tumor cells with alternative activation of AR signaling. The current more intensive androgen deprivation treatments in CRPC, including abiraterone or enzalutamide, may thereby further select for gain-of-function *AR* mutations to allow the restoration of AR signaling in tumor cells. Recent studies on PCa cell lines and patient samples have revealed multiple AR point mutations, including treatment-induced F877L and T878A [8, 10, 29, 30]. The majority of these mutations occur within the LBD domain and function to alter the ligand binding specificity of AR. Another type of treatment-induced *AR* alteration is the increased expression of *AR* splice variants, including *AR-V7* or *V12*, which can constitutively activate transcription of AR-regulated genes despite the level of ligands [14–16, 21]. Multiple studies have reported direct or indirect mechanisms that drive the increased expression of these variants in CRPC or abiraterone/enzalutamide-resistant PCa [18, 19, 31]. In this study, through examination of tumor samples from patients relapsed for the treatment of ketoconazole and dutasteride, we have discovered a novel nonsense mutation Q784* at AR LBD (exon 6), which represents a distinct class of *AR* mutations that produces a partially LBD-truncated AR protein that structurally and functionally mimics certain LBD-truncated AR splice variants. Interestingly, unlike constitutively activated AR-V7 or V12, this mutant appeared to be a loss-of-function mutation when we studied its transcriptional activity in AR negative cells (see Figure 2). Although many previously reported AR variants (such as AR-V1 or mAR-V2) [21] also deficiently drive transcription, this AR-Q784* mutant mechanistically differs from those variants. It still harbors an intact nuclear localization signal (NLS) but loses the nuclear export signal (NES), and thereby is constitutively present in the nucleus and binds to chromatin (see Figure 3), whereas many LBD truncated AR variants localize cytoplasmically due to loss of NLS, which spans exon3/4 of AR [26, 27]. Most AR variants with exon 3 truncation (except for AR-V7 due to its unique C-terminal sequence) localize cytoplasmically while other AR variants that are truncated after exon 4 (such as AR-V12) should retain intact NLS and localize to the nucleus. Loss of NES (within LBD of AR) in addition to retaining NLS also appeared to be essential to determine the nuclear localization of such AR truncations (see Figure 4). The loss of the transcriptional activation function of AR-Q784* may be due to the remaining fraction of LBD that may impair the N-terminal AF-1 function. However, the precise mechanism on this repressive molecular interaction remains to be determined in the future study. Nonetheless, the constitutive DNA binding ability of this mutant may allow it to function as a co-regulator of AR-FL and enhance the transcriptional activity of AR-FL. This is consistent with our studies demonstrating that AR-Q784* can dimerize with AR-FL and enhance its transcriptional activity, and that LNCaP cells stably expressing AR-Q784* exhibit enhanced chromatin binding of endogenous AR-FL and recruitment of p300 coactivator, thereby increasing AR-FL activity under low ligand conditions (see Figure 5, 6, 7). However, the precise mechanisms on how AR-Q784* interacts with AR-FL at AREs and how their interactions affect coactivator recruitments remain to be determined in the future studies.

Similar LBD-truncation mutations of *AR* including nonsense or point deletion (induce frame-shift) mutations were previously identified as germ line mutations from patients with androgen insensitive syndrome, indicating this class of *AR* mutations are generally loss-of-function mutations (*AR* is located at X-chromosome) [32]. This is consistent with our finding that AR-Q784* is incapable of activating transcription by itself. Interestingly, a few nonsense or point deletion mutations at AR LBD region were found in localized PCa tumors [33, 34] although *AR* mutations are generally rare in primary PCa. The expression of these mutations may not necessarily indicate loss of AR signaling since PCa cells can still harbor *AR-FL* allele due to the abnormality of chromosome numbers caused by chromosome instability. However, these mutations are likely passenger mutations due to the high-level androgen condition in primary PCa that can fully activate AR-FL. Importantly, the frequency of point mutations and gene amplifications of *AR* is substantially increased in CRPC samples [24], indicating that the ADTs may select for additional chromosome alterations in order to restore AR signaling. Therefore, these LBD-truncation mutations may be further selected and are functionally important in driving PCa resistance to the standard or more aggressive ADTs. Indeed, a similarly structured somatic nonsense mutation (R787*) has been identified from metastatic CRPC samples, suggesting this class of mutations may contribute to the restoration of AR signaling in CRPC [35]. Although the functional significance of these mutations on PCa progression has not been determined, based on our findings we speculate that they may play a similar role as Q784* mutant to enhance wildtype AR activity when ligands are limited.

In summary, we highlight in this study the biological functions of a distinct class of AR variations, including nonsense or point-deletion mutations within LBD and certain splice variants (such as exon 6/7/8 escaping variants) that only lose a fraction of LBD domain (with disrupted NES), in CRPC or CRPC resistant to CYP17 inhibitor treatments. This class of mutants/variants are transcriptionally deficient but can enhance the activity of AR-FL stimulated by low level of androgen ligands possibly due to their constitutive DNA binding ability and dimerization with AR-FL. This function is consistent with many AR splice variants (such as V7 or V12) that can dimerize with AR-FL and enhance its activity in CRPC [21–23]. However, this class of AR variations functionally differ from the constitutively active AR-V7 or V12 variants because heterodimerization with AR-FL is absolutely required for them to restore AR signaling. Although these partially LBD-truncated mutations were relatively rare in current studies of CRPC, we speculate that they will be more frequently found in CRPC patients who eventually fail abiraterone therapy. Nonetheless, as we indicated in this study, the activity of this class of AR mutants relies on AR-FL and thus can be fully prevented by antagonist treatments such as enzalutamide. Therefore, this study provides an additional rationale for treating PCa patients with combination therapy of abiraterone and enzalutamide (such therapies are currently tested in multiple clinical trials) in order to prevent the emergence of this class of *AR* mutations.

## MATERIALS AND METHODS

### Plasmids and stable cell lines

Expression vectors for pcDNA3.1(+), HA-tagged AR-FL (pcDNA3.1(+)-kozac-HA-HA vector backbone), and FLAG-tagged AR-FL (p3xFLAG-CMV-10 vector backbone) were kindly gifted from Drs. S. Chen and S. Balk at Beth Israel Deaconess Medical Center. AR-Q784* point mutation (C->T) and AR-K823* point mutation (A->T) were generated using QuickChange Lightning Site-Directed Mutagenesis Kit (Agilent Technologies Cat. # 210518) per the manufacturer's protocol. The primers for generating AR-Q784* are: forward, 5’-CT CATTCGGACACACTAGCTGTACATCCGGGAC-3’; reverse, 5’-GTCCCGGATGTACAGCTAGTGTGTCC GAATGAG-3’. The primers for generating AR-K823* are: forward, 5’-TTCATCAAAGAATTTTTGATTTTACAGCCCATCCACTGGAATAATG-3’; reverse, 5’-CATTATTCCAGTGGATGGGCTGTAAAATCAAAAATTCTTTGATGAA-3’. PLIX lentiviral vectors expressing AR-FL or AR-Q784* mutants were generated using the Gateway Technology with Clonase II (Invitrogen Cat #. 12535-029). Lenti-virus carrying PLIX-AR-FL or PLIX-AR-Q784* were assembled in HEK293T cells. LNCaP cells were then infected with lenti-virus in the presence of polybrene, followed by selection with puromycin.

### Cell culture

LNCaP cells were cultured in RPMI-1640 with 10% FBS (fetal bovine serum). PC-3 cells were in F-12K medium with 10% FBS. COS-7 cells were in DMEM with 10% FBS. Stable LNCaP cell lines expressing empty vector, AR-FL, and AR-Q784* were maintained in tetracycline-free medium (RPMI-1640 supplemented with 10% tetracycline-screened FBS). For androgen stimulation assays, cells were grown to 50-60% confluence in hormone-depleted medium (phenol red-free RPMI-1640 medium with 5% charcoal/dextran stripped FBS) for 2 days prior to treatment.

### Transient transfection

For androgen stimulation assays, cells grown to 80-90% confluence in hormone-depleted medium for 24h were transiently transfected with pcDNA3.1(+) empty vector, FLAG-tagged AR-FL, HA-tagged AR-FL, FLAG-tagged AR-V7, or FLAG-tagged AR-Q784* using Lipofectamine 2000 (Invitrogen, Cat. # 11668019) for overnight, followed by 10nM DHT or other treatments for 24h.

### Luciferase reporter assay

Cells were transfected with a *Firefly* luciferase reporter construct together with a *Renilla* luciferase reporter construct for 24h prior to the treatments. Transfection was done in triplicates. *Firefly* luciferase and *Renilla* luciferase activity were measured using the Dual-luciferase Reporter Assay (Promega, Cat. # E1980) and samples were normalized for *Renilla* activities.

### Chromatin immunoprecipitation (ChIP)

For preparation of ChIP-qPCR, dispensed cells were formalin fixed, lysed, and sonicated to break the chromatin into 500–800 bp fragments, followed by immunoprecipitation. The qPCR analysis was carried out using the SYBR Green method on the QuantStudio 3 Real-time PCR system (Thermo Fisher Scientific). The primers of FKBP5 enhancer: forward, 5’-CTCTCCAACCTGCACTCCAT-3’; reverse, 5’-TAAA AGCACACAGGCGTGAA-3’. ChIP grade anti-AR (N-terminal) and anti-AR (C-terminal) were purchased from Santa Cruz, and anti-p300 was purchased from Abcam.

### Immunoprecipitation (IP)

Cells were harvested in triton lysis buffer containing protease inhibitors. The protein samples were then incubated with covalently immobilized anti-HA containing agarose beads (Sigma), followed by elution of IP-targets. The eluted proteins were then subjected to immunoblotting.

### RT-PCR and immunoblotting

RNA was extracted with TRIzol Reagent (Invitrogen Cat. # 15596018) based on manufacturer's protocol. The gene expression was measured using real-time RT-PCR analyses with Taqman one-step RT-PCR reagents on the QuantStudio 3 Real-time PCR system (Thermo Fisher Scientific) and results were normalized to co-amplified GAPDH. The primers and probes are listed below, *PSA*: forward, 5′-GATGAAACAGGCTGTGCCG-3′; reverse, 5′-CCTCACAGCTACCCACTGCA-3′; probe, 5′-FAM-CAGGAACA AAAGCGTGATCTTGCTGGG-3′, FKBP5 primers/probe mix was directly purchased from Thermal Fisher Scientific (assay ID: Hs01561006_m1 FKBP5). For immunoblotting, cell lysates were collected by boiling in 2%SDS for 15 minutes and anti-AR-(N-terminal) (for detection of total AR), anti-AR-(C-terminal) (for detection of AR-FL only) (Santa Cruz), anti-FLAG, anti-HA (Sigma), anti-LaminB1, or anti-GAPDH (Abcam) antibodies were used for blotting. Gels shown are representative of at least 3 independent experiments.

### Cell fractionation assay and immunofluorescence staining

COS-7 cells were transfected with AR-FL, AR-Q784*, or AR-K823*. NE-PER Nuclear and Cytoplasmic Extraction Reagents (Thermo Scientific Cat. # 78833) were used to extract nuclear and cytoplasmic proteins following manufacturer's protocol. Immunoblot analysis was then performed as described above. For immunofluorescence staining, cells were fixed with methanol and then incubated with AR-441 antibody (Santa Cruz), followed by incubation with fluorescence labeled secondary antibodies (Alexa Fluor 488, Molecular Probes). Micrographs were taken under EVOS auto fluorescence microscope (ThermoFisher) at the same microscope settings.

### Flow cytometry

Cell proliferation assay was performed on an EMD Millipore Muse^TM^ Cell Analyzer using its Count & Viability Kit. Briefly, LNCaP stable cell lines were maintained in hormone-depleted RPMI medium (5% hormone-depleted FBS) and seeded in 6-well plates at 3 × 10^5^ cells per well with same medium. After treatment, cells were collected and washed with PBS, followed by mixing with Muse^TM^ Count & Viability Reagent. After 5 min incubation, cells were subjected to counting using Cell Analyzer.

### Statistical analysis

Data in bar graphs represent mean±SD of at least 3 biological repeats. Statistical analysis was performed using Student's *t*-test by comparing treatment versus vehicle control or otherwise as indicated. p-Value<0.05 (*) was considered to be statistically significant. Results for immunoblotting are representative of at least three experiments.
